# Study on the Relationship Between Orthostatic Hypotension and Heart Rate Variability, Pulse Wave Velocity Index, and Frailty Index in the Elderly: A Retrospective Observational Study

**DOI:** 10.3389/fcvm.2020.603957

**Published:** 2020-11-27

**Authors:** Lun Li, Huanhuan Li, Li He, Hongyan Chen, Yunqiao Li

**Affiliations:** ^1^Department of Cardiology, Tongji Medical College, Wuhan Fourth Hospital, Puai Hospital, Huazhong University of Science and Technology, Wuhan, China; ^2^Department of Physical Examination, Tongji Medical College, Wuhan Fourth Hospital, Puai Hospital, Huazhong University of Science and Technology, Wuhan, China; ^3^Department of Geriatrics, Tongji Medical College, Union Hospital, Huazhong University of Science and Technology, Wuhan, China

**Keywords:** orthostatic hypotension, heart rate variability (HRV), pulse wave velocity (PWV), ankle brachial pressure index (ABI), FI

## Abstract

**Background:** Orthostatic hypotension (OH) is a common disease of the elderly. It is generally believed that the pathogenesis of OH is related to the impairment of autonomic nerve function and the decreased vascular capacity regulation. This study aims to explore the relationship between OH and heart rate variability (HRV) parameters, which reflects autonomic nerve function; ankle-brachial pressure index (ABI), which reflects the degree of vascular stenosis; pulse wave velocity (PWV) index, which reflects vascular stiffness; and frailty index (FI), which reflects the overall health status of the elderly.

**Methods:** From January to September 2018, 24-h HOLTER monitoring, PWV, and ABI were performed in 108 elderly patients with OH and 64 elderly patients who underwent physical examination in our hospital. Analysis software was used to record the subject's standard deviation of the cardiac cycle (SDNN), the standard deviation of the 5-min average cardiac cycle (SDANN), the square root of the average square sum of consecutive n-interval differences (rMSSD), the percentage of the number of adjacent cardiac interval differences >50 ms (pNN50), low frequency (LF), high frequency (HF), very low frequency (VLF), and low frequency/high frequency ratio (LF/HF). Then, FI was evaluated qualitatively and quantitatively in the form of a scale.

**Results:** There was no statistical difference between the two groups on the basis of age, sex, body mass index (BMI), low-density lipoprotein (LDL), resting heart rate, blood pressure, fasting blood glucose, long-term medication, etc. There were significant differences in PWV, SDNN, LF, VLF, and LF/HF between the two groups (*P* < 0.05). The risk factor of OH in the qualitative (*P* = 0.04) and quantitative (*P* = 0.007) index FI was higher in the OH group than in the control group. The risk factors of OH were PWV, SDNN, VLF, LF/HF, and FI, where FI was positively correlated and LF/HF was negatively correlated.

**Conclusions:** The pathogenesis of OH is related to vascular stiffness, imbalance of autonomic nerve regulation, and its comprehensive health status in the elderly. However, arteriosclerosis has not been confirmed as an independent risk factor.

**Clinical Trial Registration:** Retrospectively registered, http://www.chictr.org.cn.

## Background

Orthostatic hypotension (OH) is a common disease status of the elderly. Its incidence increases with age. According to American statistics, the prevalence rate of the community population aged over 65 was about 20–30% (1). In the Irish elderly population study, the prevalence of OH was 41% among people over 80 years old (2). OH is considered to be the third most common cause of syncope, second only to vasovagal syncope and cardiac syncope (3), so patients have an increased risk of falling, with complications including fractures, infections, and reduced mobility (4). Some studies showed that OH was positively correlated with the overall prevalence of dementia and may also lead to Alzheimer's disease and vascular dementia (5). Clinical research showed that the prognosis of OH patients was poor, which could increase all-cause mortality by 1.5 times in a 5-years follow-up (6).

At present, there are some studies on the pathogenesis of OH. Some studies have shown that non-neurogenic OH was related to autonomic nervous dysfunction and decreased vascular capacity regulation (7). Clinically related treatments of OH also focus on improving autonomic nerve function and vascular regulation (8). HRV is considered to be a well-known indicator of cardiac autonomic nerve function. Some studies also confirmed that HRV was related to OH (9, 10), but the observation parameters were inconsistent, the mechanism theory was not rigorous, and there were also contrary research results (11, 12).

Based on the mechanism of decreased vascular volume regulation, combined with the characteristics of elderly patients, we speculated that there might be a correlation between atherosclerosis and OH, but there were few relevant reports. In consideration of the elderly with multiple mental disorders, we also tried to explore the relationship between the comprehensive state of the elderly and OH. Accordingly, we designed this study.

## Methods

### Participants

From January to September 2018, 108 cases of OH elderly patients from the Geriatrics Department and Cardiology Department in the eastern district of Wuhan Puai Hospital were screened as observation groups (OH group). In the same period, 64 cases of elderly people from the Physical Examination Department in the eastern district of Wuhan Puai Hospital were taken as control group (CON group). Strobe statements were used for study identification, screening, and inclusion.

#### Selection Criteria

(1) Age ≥65 years old, able to cooperate with researchers; (2) In the stable stage of the disease, stop intravenous infusion for more than 3 days; (3) OH group meets diagnostic criteria (13), with or without related clinical symptoms; CON group excluded OH diagnosis; (4) To obtain informed consent.

#### Exclusion Criteria

(1) Atrial fibrillation or obvious arrhythmia; (2) Pacemaker implantation; (3) Diabetes; (4) Drugs, such as antidepressants, anti-Parkinson, anti-psychotic, Raas blockers, and Beta blockers that have a significant effect on autonomic nerve function are being taken; (5) At the stage of disease onset, needs clinical treatment, such as intravenous infusion; (6) Those who have not obtained informed consent and cannot cooperate.

### Study Design

#### OH Diagnostic Criteria

Refer to the definition in the expert consensus issued by the American Institute of Autonomous Neurology and Neuropathy in 1996: The blood pressure was measured every minute within 3 min after the change from lying position to standing position. The systolic and/or diastolic blood pressure decreased ≥20 mmHg and/or diastolic blood pressure decreased ≥10 mmHg within 3 min (13).

#### Determination and Entry of the OH Group

From January to September 2018, elderly OH patients from the Geriatrics Department and Cardiology Department of Wuhan Puai Hospital (age ≥65) were screened by the researchers under the condition of stable preexisting disease. The method was as follows: After the patient stayed in bed for 15 min, the researcher used a calibrated mercury sphygmomanometer to measure the blood pressure and heart rate of the lying humeral artery and recorded it. The patient's blood pressure and the heart rate of the same brachial artery were measured every minute after standing upright and recorded to 3 min after standing upright. Patients with reduced systolic pressure ≥20 mmHg and/or diastolic pressure ≥10 mmHg within 3 min were diagnosed as OH. After informed consent was obtained, a total of 108 cases entered the group and completed the study (*n* = 108).

#### Entry of the Control Group

From January to September 2018, the elderly (age ≥65 years old) who came to the Physical Examination Department of Wuhan Puai Hospital for physical examination were enrolled by the researchers after OH detection. Ruling out OH diagnosis and obtaining informed consent, a total of 64 cases were enrolled and completed the study (*n* = 64).

#### Observation Indicators Collection

The two groups of subjects who were selected for registration both recorded general data, body mass index (BMI), resting blood pressure, heart rate, and medication. The following routine biochemical tests were performed: blood fat, blood sugar, improved ankle-brachial pressure index (ABI), pulse wave velocity (PWV), and 24-h HOLTER tests. Qualitative and quantitative scores of “frailty” were evaluated and recorded by a specially designated researcher. All data were reviewed and verified by two researchers independently.

##### Collection method of arteriosclerosis parameters

The ABI and PWV were measured using the VP-1000 automatic arteriosclerosis tester of OMRON Colin of Japan. Resting at least 5 min before the test, all subjects lay on the diagnostic bed on their backs, with the cuff tied to the upper and lower limbs in accordance with the operating rules. The upper arm sleeve belt air bag marked the brachial artery and the lower edge of the cuff was 2–3 cm from the elbow socket. The lower extremity cuff airbag mark was aligned with the inner side of the lower extremity, and the lower edge of the cuff was 1–2 cm from the inner ankle. The cuff was loosened moderately. The electrocardiogram (ECG) sensor was placed in the second left rib, body height parameters were input, the instrument automatically measured ABI and calculated PWV, and the researchers recorded the data.

##### Heart rate variability indicators collection method

All subjects were monitored using 24-h HOLTER, the device was GE Seer Light Record box, and the heart rate variability (HRV) analysis software used was GE MARS Software. HRV analysis was based on the guidelines of the European College of Cardiology and the North American Pacemaker and Electrophysiological Society. NN intervals were RR intervals in sinus rhythm, and average NN intervals were the mean value of RR intervals in sinus rhythm. Intervals greater than or <30% of the average of the first four intervals are considered artifacts and removed from the analysis record. According to the automatic editing program, each ECG record was manually reviewed by two authors blinded to avoid abnormal QRS complex morphology, ectopic heartbeat, and motion artifacts, and to ensure that the HRV analysis program correctly marks the R wave, so as to accurately detect the R–R interval. Make corrections if necessary. According to the measurement standard, the following HRV measurements were calculated in the time domain and frequency domain (14).

Time-domain related parameters:

- The standard deviation of normal-to-normal RR interval (SDNN), in ms.
- The standard deviation of the 5-min average cardiac cycle (SDANN), in ms.
- The square root of the average square sum of consecutive n-interval differences (rMSSD), in ms.
- The percentage of the number of adjacent cardiac interval differences >50 ms (pNN50), in%.

Frequency-domain related parameters:

For frequency-domain HRV analysis, RR time series were interpolated at 250 ms to obtain isometric values. Using fast Fourier transform (FFT), we quantified HRV in four frequency bands. Three characteristic frequencies were identified: LF, HF, and VLF when analyzing 2- to 5-min ECG recordings. ULF bands are identified with VLF bands in a 24-h recording.

- Low frequency (LF) band (LF power in the range of 0.04–0.15 Hz), in ms^2^.
- High frequency (HF) band (HF power in the range of 0.15–0.40 Hz), in ms^2^.
- Very low frequency (VLF) band (VLF power in the range of 0.003–0.04 Hz), in ms^2^.
- Ultra low frequency (ULF) band (ULF power in the range of 0.0001–0.003 Hz), in ms^2^.
- We also considered the LF/HF ratio.

##### Qualitative frailty index (FI) evaluation and collection

In this study, the Groningen frailty indicator (GFI) scale (15) was selected as the research object for qualitative evaluation of frailty status. This scale was put forward by the Dutch scholar Peters in 2010. It presupposed 15 indexes in four fields: physical ability, cognition, society, and psychology (Supplementary Table 1). The subjects of each index only need to select “yes” or “no” (“yes” means that the item cannot be completed independently, score 1 point; “no” means that the item can be completed independently without the help of others, score 0 point); the cumulative score value is 0–15 points; if the result is ≥4 points, it is recorded as “frailty”; if <4 points, it is recorded as “healthy.” In this study, the scale was completed and recorded by fixed researchers at the time of recruitment.

##### Quantitative FI formulation

The quantitative index of frailty adopted the currently recognized FI proposed by Rockwood et al. (16). FI is the accumulation of multiple indicators based on diseases, activities of daily life, clinical evaluation results, etc., which can assess the risk of adverse health outcomes and can objectively evaluate each individual independent of age and functional status.

###### Quantitative FI evaluation

List the common health deficits reflecting frailty that met the standards and with a score of 0–1 for each item. Each defect can be determined as multiple values according to the situation; for example, there are good, medium, and bad conditions, and the score can be set as 0, 0.5, and 1. You need 4 values to give (0, 0.33, 0.67, 1.0) four scores. The corresponding individual situation was scored. The FI score was the ratio of the individual's current number of deficits score to the total number of deficits score. For example, consider that a health project data set had 50 variable items, each of which met the criteria considered to be a health deficit. If an individual did not have one, his FI will be 0/50 = 0 (theoretically, this situation did not exist). If an individual had 10 deficits of 1 point, the FI index would be 10/50 = 0.20.

###### Quantitative FI project identification and collection

According to the inclusion criteria of the FI project (17), referring to relevant literature (18) and our actual situation, we established 30 FI indicators in six aspects: 6 physiological self-care capabilities (needed help in cooking, shopping, housework, bathing, urine control, and outdoor walking; one score of 1 point; otherwise, 0 points); 1 cognitive function (MMSE scale score, <10 points for 1 point, 10–25 points for 0.5 points, >25 points for 0 points); 16 cases of illness (allergies other than food, asthma, degenerative joints, rheumatism, hypertension, diabetes, migraines, chronic bronchitis, sinusitis, epilepsy, heart disease, tumors, gastrointestinal diseases, cerebrovascular diseases, Alzheimer's disease, or other dementia, cataracts; one score of 1 point; otherwise, 0 points); 4 health self-assessments [hearing impairment affected life, visual impairment affected life, serious illness in the past year (reported by a doctor to be seriously ill or critically ill), and 4 or more prescription drugs; one score of 1 point; otherwise, 0 points]; 2 mental health items (often consciously nervous or depressed, not interested in things; one score of 1 point; otherwise, 0 points); and 1 item (non-conscious; the weight dropped by 5 kg in the past year; one score of 1 point, no score of 0 points), for a total of 30 points. Dividing the individual score by the total score of 30 points yields the individual's FI (Supplementary Table 2). This indicator was collected and scored by specialized researchers.

### Statistical Analysis Methods

Quantitative data were expressed as mean ± SD, and qualitative data were expressed as the number of cases (percentage). The normal distribution of continuous variables was tested by Kolmogorov–Smirnov. The variance homogeneity test used Levene test. The continuous data of the normal distribution were evaluated by an independent-samples *t*-test or a Bonferroni single-factor variance analysis (one-way ANOVA). The percentage of the comparison classification variable was tested using chi square. We determined the relevant risk factors for OH by the multiple logistic regression model. All statistical analysis was performed using SPSS 22.0 software. *P* < 0.05 was statistically significant.

## Results

### Study Selection

**Figure 1:**
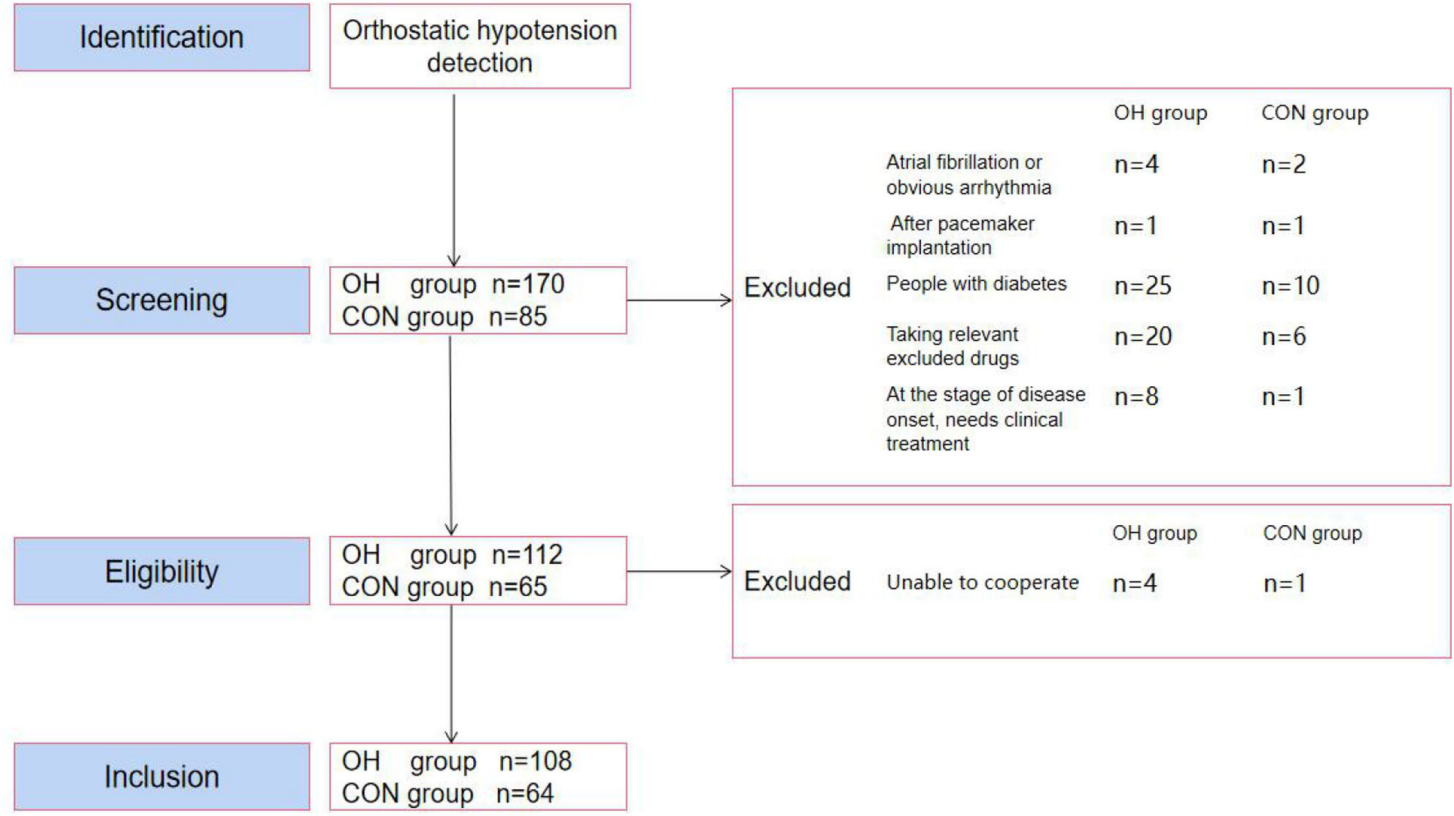
Participant selection flow diagram. OH, orthostatic hypotension group; CON, control group.

The Medsci sample size tools (MSST) was used to calculate the sample size. According to the matching ratio of 2:1 in the OH group and the CON group, a total of 255 study populations were included according to the results of the OH test. Through the relevant exclusion screening criteria, a total of 78 people failed. Subsequently, five more people were not eligible for the study because they could not cooperate with the experiment. In the end, 172 people entered the study (Figure 1). The steps used followed the Strobe statement.

### General Characteristics of Participants

There was no significant difference between the two groups in age, gender, smoking, drinking wine, BMI, resting blood pressure, resting heart rate, hypertension, hyperlipidemia, hyperuricemia, tumors, and cerebral infarction. There was no significant difference between the two groups in laboratory biochemical indexes, such as fasting blood sugar and LDL (Table 1).

**Table 1:** General characteristics of participants.

	**OH Group** **(*n* = 108)**	**CON Group** **(*n* = 64)**	***P*-value**
Age (years)	72.89 ± 4.48	72.27 ± 3.92	*P* = 0.12
Gender (M/F)	57/51	31/33	*P* = 0.63
Smoking	34 (30%)	17 (27%)	*P* = 0.50
Drinking wine	27 (25%)	13 (20%)	*P* = 0.48
BMI	23.84 ± 1.46	23.73 ± 1.59	*P* = 0.49
Systolic pressure (mmHg)	134.17 ± 13.79	131.28 ± 12.56	*P* = 0.85
Diastolic pressure (mmHg)	71.67 ± 10.24	71.16 ± 8.21	*P* = 0.08
Static heart rate (bpm)	70.33 ± 7.34	70.69 ± 7.54	*P* = 0.24
Fasting blood sugar (mmol/l)	4.79 ± 0.58	4.69 ± 0.52	*P* = 0.32
LDL (mmol/l)	2.81 ± 0.67	2.68 ± 0.60	*P* = 0.12
Hypertension	31/108 (29%)	20/64 (31%)	*P* = 0.72
Hyperlipidemia	63/108 (58%)	29/64 (45%)	*P* = 0.10
Hyperuricemia	18/108 (17%)	9/64 (14%)	*P* = 0.65
Tumors	8/108 (7%)	3/64 (5%)	*P* = 0.48
Cerebral infarction	27/108 (25%)	12/64 (19%)	*P* = 0.34

### General Characteristics of Participants Logistic Regression Analysis

Taking OH as the dependent variable and using age, gender, smoking, drinking wine, BMI, resting blood pressure, resting heart rate, fast blood sugar, LDL hypertension, hyperlipidemia, hyperuricemia, tumors, and cerebral infarction as independent variables, logistic regression analysis showed that the above indicators were not the risk factors of OH (Table 2).

**Table 2:** General characteristics of participants logistic regression analysis.

	***B***	**S.E.**	**Wald**	***df***	***P-*value**	**Exp** **(*B*)**	**95% CI for EXP(*****B*****)**	
							**Lower**	**Upper**
Age (years)	−0.045	0.429	0.011	1	0.917	0.956	0.413	2.215
Gender (M/F)	0.036	0.044	0.655	1	0.418	1.036	0.951	1.129
Smoking	−0.331	0.772	0.184	1	0.668	0.718	0.158	3.260
Drinking wine	−0.179	0.771	0.054	1	0.816	0.836	0.184	3.792
BMI	−0.003	0.112	0.001	1	0.981	0.997	0.801	1.242
Systolic pressure (mmHg)	0.017	0.015	1.364	1	0.243	1.017	0.988	1.047
Diastolic pressure (mmHg)	0.024	0.021	1.312	1	0.252	1.024	0.983	1.067
Static heart rate (bpm)	−0.027	0.025	1.117	1	0.291	0.974	0.926	1.023
Fasting blood sugar (mmol/l)	0.272	0.313	0.754	1	0.385	1.312	0.711	2.422
Low-density lipoprotein (mmol/l)	0.172	0.312	0.304	1	0.581	1.188	0.644	2.191
Hypertension	0.398	0.388	1.054	1	0.305	1.489	0.696	3.185
Hyperlipidemia	−0.444	0.380	1.366	1	0.243	0.641	0.304	1.351
Hyperuricemia	−0.119	0.476	0.063	1	0.802	0.888	0.349	2.256
Tumors	−0.688	0.740	0.865	1	0.352	0.502	0.118	2.142
Cerebral infarction	−0.426	0.417	1.047	1	0.306	0.653	0.289	1.477

*B, coefficient; S.E., standard error; Wald, card square; df, degree of freedom; OR, odds ratio*.

### Analysis of Defined Daily Dose (DDD) and Usage of Antihypertensive Drugs and Logistic Regression Analysis

The results showed that there were no significant differences in the DDD of antihypertensive drugs, calcium channel antagonists, alpha receptor antagonists, diuretics, and statins between the two groups (Table 3). Taking OH as the dependent variable and the above indexes as the independent variable, logistic regression analysis showed that the above indexes were not the risk factors of OH (Table 4).

**Table 3:** Analysis of DDD and usage of antihypertensive drugs.

**Drug use**	**OH Group** **(*n* = 108)**	**CON Group** **(*n* = 64)**	***P-*value**
DDD	2.3 ± 3.3	2.5 ± 2.4	*P* = 0.65
Calcium channel antagonists	27/108 (25%)	19/64 (30%)	*P* = 0.51
Alpha receptor antagonists	11/108 (10%)	4/64 (6%)	*P* = 0.38
Diuretic	15/108 (14%)	7/64 (11%)	*P* = 0.64
Statin	60/108 (56%)	31/64 (48%)	*P* = 0.43

*OH, orthostatic hypotension group; CON, control group*.

**Table 4:** DDD and usage of antihypertensive drugs logistic regression analysis.

	***B***	**S.E.**	**Wald**	***df***	***P*-value**	**Exp** **(*B*)**	**95% CI for EXP(*****B*****)**	
							**Lower**	**Upper**
DDD	−0.056	0.076	0.541	1	0.462	0.946	0.816	1.097
Calcium channel antagonists	0.198	0.453	0.190	1	0.663	1.219	0.501	2.963
Alpha receptor antagonists	−0.771	0.719	1.148	1	0.284	0.463	0.113	1.895
Diuretic	−0.298	0.589	0.256	1	0.613	0.742	0.234	2.354
Statin	−0.379	0.326	1.350	1	0.245	0.685	0.361	1.297

*B, coefficient; SE, standard error; Wald, card square; df, degree of freedom; OR, odds ratio*.

### The Results of the Atherosclerosis Test

**Figure 2:**
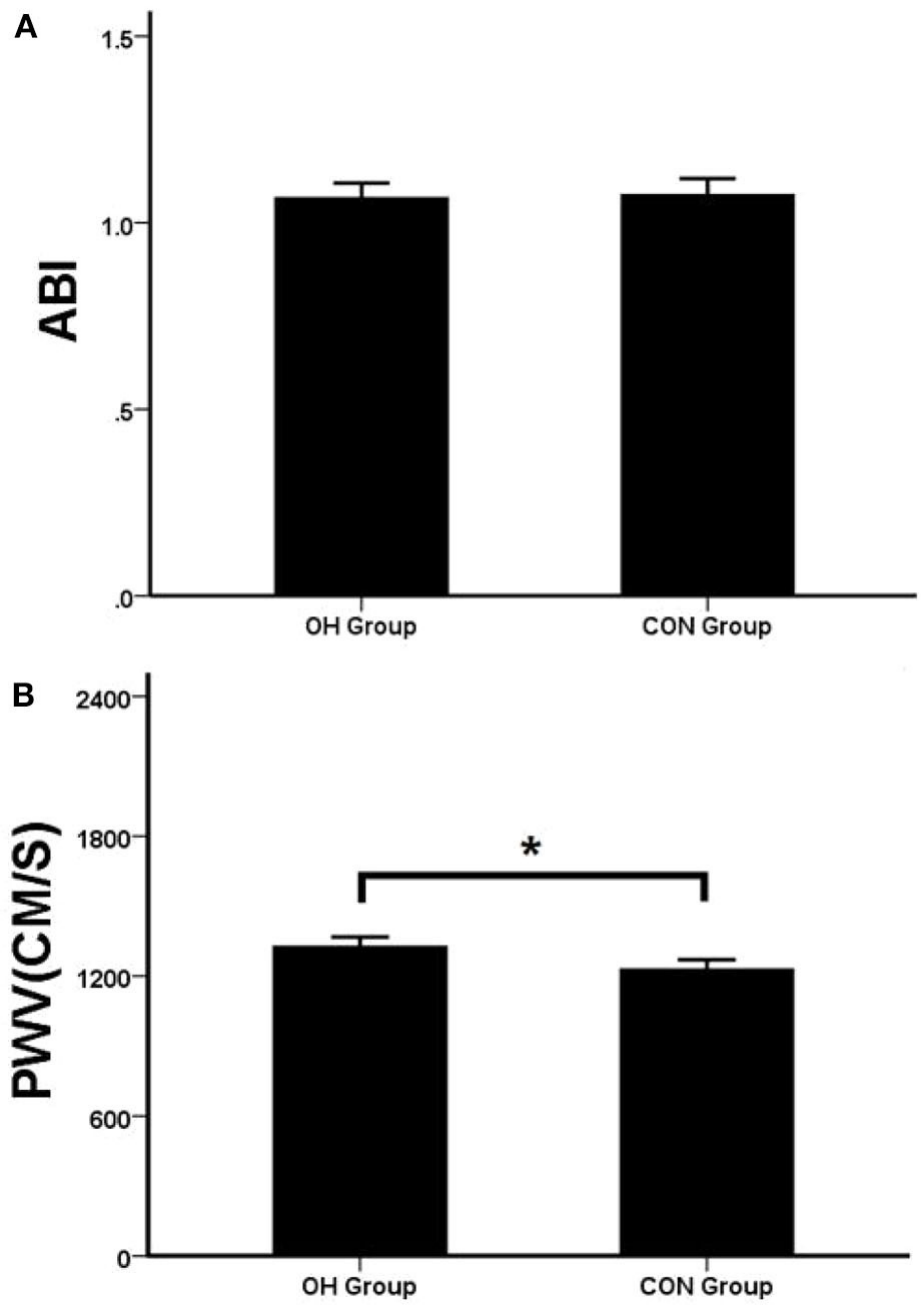
**(A)** The two groups of ABI. **(B)** The two groups of PWV. OH, orthostatic hypotension group; CON, control group. *P < 0.05, statistical difference.

The results indicated that there was no statistical difference about ABI between the two groups (*P* = 0.14) (Figure 2A). PWV was significantly higher in the OH group than in the control group (*P* = 0.035) (Figure 2B).

### The Results of 24-h HOLTER Tests

**Figure 3:**
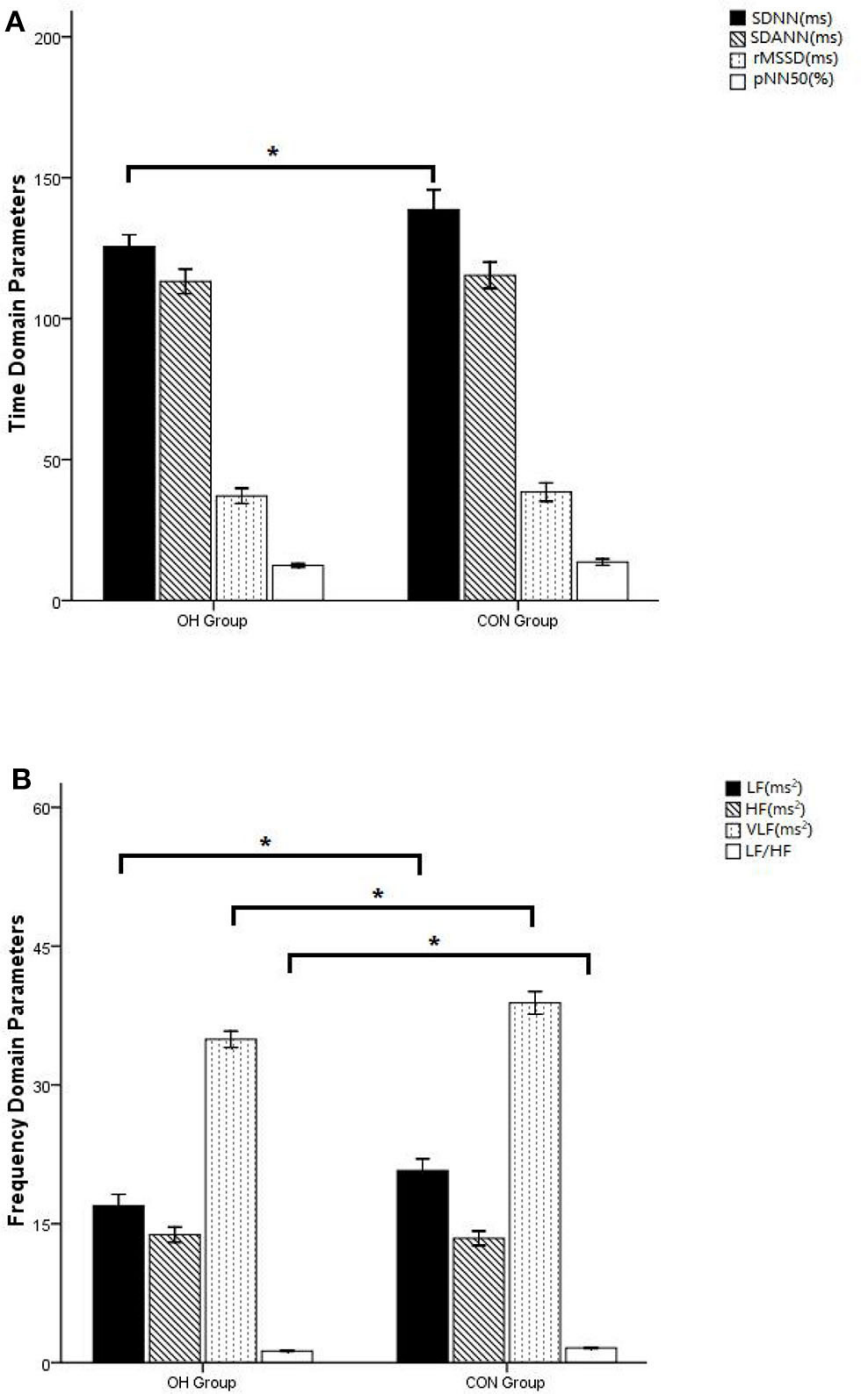
**(A)** Comparison of time domain indicators of heart rate variability. **(B)** Comparison of heart rate variability frequency domain indicators. OH, orthostatic hypotension group; CON, control group. *P < 0.05, statistical difference.

In the detection of HRV time-domain-related parameters, there was no statistical difference of SDANN, rMSSD, and pNN50 between the two groups (*P* > 0.05). Compared with the CON group, SDNN in the OH group decreased significantly (*P* < 0.05) (Figure 3A). In the detection of HRV frequency-domain-related parameters, there was no statistical difference of HF between the two groups (*P* > 0.05). Compared with the CON group, LF, VLF, and LF/HF decreased significantly in the OH group (*P* < 0.05) (Figure 3B).

### Qualitative and Quantitative Results of FI

**Figure 4:**
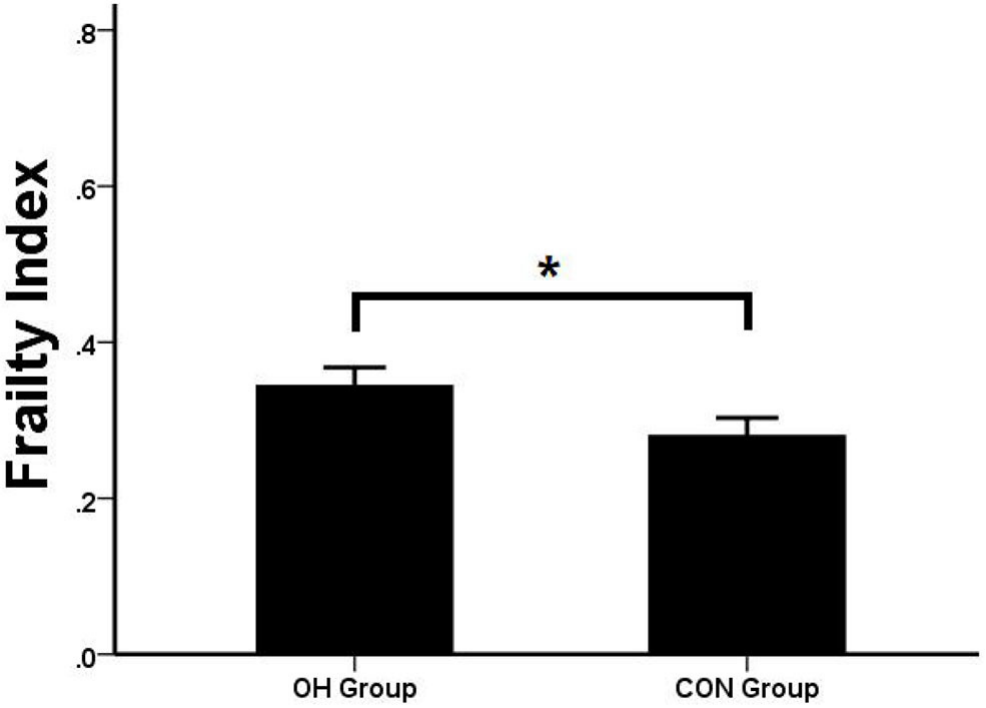
Comparison of FI. OH, orthostatic hypotension group; CON, control group. *P < 0.05, statistical difference.

According to the qualitative results of FI, the proportion of compliance with frailty in the OH group was significantly higher than that in the control group (*P* = 0.04) (Table 5). The quantitative study of FI showed that the OH group was higher than that of the control group, and there was significant statistical difference between the two groups (*P* = 0.007) (Figure 4).

**Table 5:** Comparison of qualitative frailty.

	**OH Group** **(*n* = 108)**	**CON Group** **(*n* = 64)**	***P*-value**
Qualitative frailty (Y/N)	60/48 (55.6%)	25/39 (39.1%)	*P* = 0.04^*^

*OH, orthostatic hypotension group; CON, control group*.

* *P < 0.05, statistical difference*.

### The Relevant Risk Factors for OH

Taking OH as the dependent variable and using ABI, PWV, SDNN, SDANN, rMSSD, pNN50, LF, HF, VLF, LF/HF, and FI as independent variables, logistic regression analysis showed that PWV, SDNN, VLF, LF/HF, and FI were all predicted factors of OH. Among them, PWV and FI were positively correlated, and SDNN, VLF and LF/HF were negatively correlated. FI and LF/HF were the most affected (Table 6).

**Table 6:** Logistic regression results of the relevant risk factors.

	***B***	**SE**	**Wald**	***df***	**Sig.**	**Exp** **(*B*)**	**95% CI for EXP(*****B*****)**	
							**Lower**	**Upper**
ABI	−1.773	1.281	1.914	1	0.167	0.170	0.014	2.094
PWV	0.002	0.001	3.983	1	0.046^*^	1.002	1.000	1.004
SDNN	−0.021	0.008	6.398	1	0.011^*^	0.979	0.963	0.995
SDANN	0.011	0.011	1.167	1	0.280	1.011	0.991	1.033
rMSSD	−0.011	0.016	0.478	1	0.489	0.989	0.958	1.021
pNN50	−0.061	0.056	1.207	1	0.272	0.941	0.843	1.049
LF	0.223	0.151	2.199	1	0.138	1.250	0.931	1.680
HF	−0.288	0.209	1.898	1	0.168	0.750	0.498	1.129
VLF	−0.588	0.181	10.493	1	0.001^*^	0.556	0.389	0.793
LF/HF	−5.898	2.471	5.695	1	0.017^*^	0.003	0.000	0.349
FI	4.440	1.968	5.091	1	0.024^*^	84.764	1.792	4009.700

*B, coefficient; SE, standard error; Wald, card square; df, degree of freedom; OR, odds ratio*.

* *P < 0.05, statistical difference*.

## Discussion

Similar to the literature (19), researchers have found that OH is a common disease state in the elderly. Some studies suggested that the onset of initial OH was related to autonomic nervous dysfunction, which was believed to be caused by the mismatch between vascular resistance and cardiac output and the active contraction of leg muscles (20). Another study showed that the cause of delayed OH was insufficient/relative insufficiency of blood volume. In the study, the patients were found to have improved syncope symptoms after using compression socks of lower limbs (21). In our daily work, researchers found that HRV abnormalities often occurred in HOLTER and ECG monitoring of OH patients. HRV involves the measurement and recording of the time interval between successive heartbeats (usually through ECG) (22). It had been proven that it reflects the activities of the autonomic nervous system (ANS) and its sympathetic and parasympathetic branches. These branches control almost all the internal organs and blood vessels (23, 24). We believed that there should be a correlation between HRV and OH. In the elderly, atherosclerosis increases with age and is related to vascular volume (25). Thus, we speculated that there might be a correlation between atherosclerosis and OH. Considering the complicated pathogenesis of OH, our observation object was the elderly, usually combined with a variety of pathophysiological conditions; we also hoped to explore the relationship between the comprehensive state of the elderly and OH. Therefore, this study established the observation object as the elderly OH patients and set the observation indexes as HRV reflecting the autonomic nerve function, ABI reflecting the degree of atherosclerotic vascular stenosis (26), PWV reflecting the degree of atherosclerotic vascular stiffness (27), and “frailty” reflecting the health status of the elderly (28).

The results showed that there was no difference in ABI between the two groups (*P* > 0.05), while PWV in the OH group was significantly higher than that in the control group (*P* < 0.05). PWV reflects the degree of vascular stiffness. It was believed that the failure of lower limbs and visceral vessels to contract in time during the body position change of OH patients was related to the damage of sympathetic adrenergic system (29), but this mechanism could not fully explain the reason that the prevalence of OH in the elderly was much higher than that in the young and middle-aged. In this study, PWV of OH patients was significantly longer than that of the control group, indicating that the vasoconstriction disorder of OH patients was also related to the increase of vascular elasticity and stiffness, which was a good supplement to the above mechanism. Moreover, in the multivariate regression model, it proved to be an independent risk factor for OH. ABI reflects the stenosis of lower extremity blood vessels. In this study, there was no significant difference between the OH group and the control group, indicating that OH had no correlation with artery stenosis.

In healthy individuals, the periodic changes of HRV were accompanied by respiratory and blood pressure fluctuations (30). Frequency domain and power spectral density (PSD) analysis using FFT analysis were used to describe the oscillations in RRi and convert them into discrete frequencies, which helped us to understand the physiological mechanism of HRV (31, 32). The time domain parameter SDNN reflects the overall situation of HRV (14). In frequency domain parameters, the periodic changes associated with arterial blood pressure (ABP) fluctuations occurred at LF of 0.10 Hz and were considered to be mediated by sympathetic nerves (33). Some experiments indicated that SDANN and HF were parasympathetic mediated (31). Through PSD analysis, the high- and low-frequency components of HRV only accounted for 5% of the total power recorded by HRV. The remaining 95% consisted of two other frequencies, called the VLF band and the ULF band (34). Recent studies had shown that the VLF band is related to changes of peripheral chemical receptor activity, thermoregulation mechanism, and fluctuation of the renin–angiotensin system (RAAS), while the ULF band is considered to reflect the oscillation caused by circadian rhythm (35). It is controversial to attribute all LF-HRV oscillations to sympathetic modulation (36, 37). Parasympathetic block also modulated the LF oscillation of HRV (38). Nevertheless, the measurement of HF-HRV and LF-HRV oscillations was considered to be a measure of sympathetic balance (33). In our experiment, we found that there was no significant difference in RMSSD and HF between the two groups, suggesting that the occurrence of OH is not related to the enhancement of parasympathetic activity. We also found that SDNN in the time domain and LF, VLF, and LF/HF in the frequency domain decreased significantly in the OH group (*P* < 0.05). In multiple regression analysis, SDNN, LF, VLF, and LF/HF were all predictors of OH (*P* < 0.05), and LF/HF was strongly correlated. It is suggested that the pathogenesis of OH is related to the imbalance of sympathetic and parasympathetic nerves.

In clinical practice, we found that the incidence of OH patients was significantly related to their age. Therefore, in this study, we specially set an indicator “frailty” to reflect the comprehensive state of the elderly, and the results were consistent with our assumption. In the qualitative index of frailty, the percentage of frailty in the OH group (55.6%) was higher than that in the control group (39.1%) and had statistical significance (*P* = 0.04). In the quantitative index of frailty, there was significant difference between the two groups (*P* < 0.05). The OH group was significantly higher than the control group, indicating that frailty was related to OH. The results of the subsequent regression equation (*P* = 0.024, OR = 84.76) also showed that FI is a predictor of OH. The relationship between them was linear and parallel.

Through this retrospective analysis, we proved that the pathogenesis of OH is related to vascular stiffness, imbalance of autonomic nerve regulation and its comprehensive health status in elder. This helps us to further understand the disease and provide us with thinking on the prevention and treatment of OH in clinical work.

### Limitations

OH is a state of disease. Its pathogenesis is complex. The observation indicators in this study are limited, and it is difficult to fully reflect its mechanism. It can only be discussed from clinical phenomena. At the same time, there are not many selected cases, and there may be errors in the conclusion. In the future, it is envisaged to formulate a more rigorous research program, conduct large-scale multi-center research, and discuss the pathogenesis of OH more accurately and comprehensively, providing evidence for clinical prevention, prediction, and treatment of OH.

## Conclusion

The pathogenesis of OH is related to arterial stiffness, imbalance of autonomic nerve regulation, and FI in the elderly. Many parameters in HRV that reflect autonomic nerve function are the predictor of OH, and the FI is closely related to OH. Indicators that reflect arteriosclerosis cannot be confirmed as an independent predictor of OH.

## Data Availability Statement

The original contributions presented in the study are included in the article/Supplementary Material, further inquiries can be directed to the corresponding author/s.

## Ethics Statement

The study was approved by the ethical committee of Puai Hospital affiliated with Huazhong University of Science and Technology, Wuhan, China (approval number: KY 2019-047-01) and conducted in accordance with the principles of the 1964 Helsinki declaration and its later amendments or comparable ethical standards. The study was registered in the Chinese Clinical Trial Register (http://www.chictr.org.cn, registration number: ChiCTR2000029090). All patients gave informed consent for participation in this study, and written consent was obtained from each participating patient.

## Author Contributions

LL and HL conceived the present study, participated in the design, conducted the data analysis, and drafted the manuscript. HL and HC collected and assembled all the data. YL and LL commented on the manuscript drafts. YL provided the material and technical support and commented on the manuscript drafts. All authors have read and approved the manuscript.

## Conflict of Interest

The authors declare that the research was conducted in the absence of any commercial or financial relationships that could be construed as a potential conflict of interest.

## Abbreviations

OH: Orthostatic hypotension
HRV: Heart rate variability
ABI: Ankle-brachial pressure index
PWV: Pulse wave velocity index
FI: Frailty index
SDNN: Standard deviation of the cardiac cycle
SDANN: Standard deviation of the 5-min average cardiac cycle
rMSSD: Square root of the average square sum of consecutive n-interval differences
pNN50: Percentage of the number of adjacent cardiac interval differences >50 ms
LF: Low frequency
HF: High frequency
VLF: Very low frequency
ULF: Ultra low frequency
LF/HF: Low frequency/high frequency ratio
BMI: Body mass index
LDL: Low-density lipoprotein
ECG: Electrocardiogram
GFI: Groningen frailty indicator
SD: Standard deviation.

## References

[1] Gupta V Lipsitz LA Orthostatic hypotension in the elderly: diagnosis and treatment. Am J Med. (2007) 120:841–7. 10.1016/j.amjmed.2007.02.023 17904451

[2] Finucane C O'Connell MD Fan CW Savva GM Soraghan CJ Nolan H Age-related normative changes in phasic orthostatic blood pressure in a large population study: findings from The Irish Longitudinal Study on Ageing (TILDA). Circulation. (2014) 130:1780–9. 10.1161/CIRCULATIONAHA.114.009831 25278101

[3] Soteriades ES Evans JC Larson MG Chen MH Chen L Benjamin EJ. Incidence and prognosis of syncope. N Engl J Med. (2002) 347:878–85. 10.1056/NEJMoa012407 12239256

[4] Berry SD Miller RR. Falls: epidemiology, pathophysiology, and relationship to fracture. Curr Osteoporos Rep. (2008) 6:149–54. 10.1007/s11914-008-0026-4 19032925 PMC2793090

[5] Min M Shi T Sun C Liang M Zhang Y Wu Y. The association between orthostatic hypotension and dementia: a meta-analysis of prospective cohort studies. Int J Geriatr Psychiatry. (2018) 33:1541–7. 10.1002/gps.4964 30247788

[6] Ricci F Fedorowski A Radico F Romanello M Tatasciore A Di Nicola M. Cardiovascular morbidity and mortality related to orthostatic hypotension: a meta-analysis of prospective observational studies. Eur Heart J. (2015) 36:1609–17. 10.1093/eurheartj/ehv093 25852216

[7] Jacob G Ertl AC Shannon JR Furlan R Robertson RM Robertson D. Effect of standing on neurohumoral responses and plasma volume in healthy subjects. J Appl Physiol (1985). (1998) 84:914–21. 10.1152/jappl.1998.84.3.914 9480952

[8] Low PA Singer W. Management of neurogenic orthostatic hypotension: an update. Lancet Neurol. (2008) 7:451–8. 10.1016/S1474-4422(08)70088-7 18420158 PMC2628163

[9] Baker J Racosta JM Kimpinski K. Comparison of heart rate variability parameters to the autonomic reflex screen in postural orthostatic tachycardia syndrome and neurogenic orthostatic hypotension. J Clin Neurophysiol. (2018) 35:115–22. 10.1097/WNP.0000000000000436 29210841

[10] Silva RMFLD Miranda CES Barbosa MT Bicalho MAC. Heart rate and its variability assessed by spectral analysis in elderly subjects with orthostatic hypotension: a case-control study. Arq Bras Cardiol. (2018) 110:303–11. 10.5935/abc.20180043 29561965 PMC5941951

[11] Lagro J Meel-van Den Abeelen A de Jong DL Schalk BW Olde Rikkert MG Claassen JA. Geriatric hypotensive syndromes are not explained by cardiovascular autonomic dysfunction alone. J Gerontol A Biol Sci Med Sci. (2013) 68:581–9. 10.1093/gerona/gls214 23070881

[12] Atli T Keven K. Orthostatic hypotension in the healthy elderly. Arch Gerontol Geriatr. (2006) 43:313–7. 10.1016/j.archger.2005.12.001 16466816

[13] Kaufmann H. Consensus statement on the definition of orthostatic hypotension, pure autonomic failure, and multiple system atrophy. Clin Auton Res. (1996) 6:125–6. 10.1007/BF02291236 8726100

[14] Heart rate variability: standards of measurement, physiological interpretation and clinical use Task Force of the European Society of Cardiology and the North American Society of Pacing and Electrophysiology. Circulation. (1996) 93:1043–65. 8598068

[15] Peters LL Burgerhof JG Boter H Wild B Buskens E Slaets JP. Measurement properties of the Groningen Frailty Indicator in home-dwelling and institutionalized elderly people. J Am Med Dir Assoc. (2012) 13:546–51. 10.1016/j.jamda.2012.04.007 22579590

[16] Rockwood K Song X MacKnight C Bergman H Hogan DB McDowell I. A global clinical measure of fitness and frailty in elderly people. CMAJ. (2005) 173:489–95. 10.1503/cmaj.050051 16129869 PMC1188185

[17] Rockwood K Mitnitski A. How might deficit accumulation give rise to frailty? J Frailty Aging. (2012) 1:8–12. 10.14283/jfa.2012.2 27092931

[18] Kojima G Liljas A Iliffe S. Frailty syndrome: implications and challenges for health care policy. Risk Manage Healthc Policy. (2019) 12:23–30. 10.2147/RMHP.S168750 30858741 PMC6385767

[19] Rutan GH Hermanson B Bild DE Kittner SJ LaBaw F Tell GS Orthostatic hypotension in older adults. The Cardiovascular Health Study. CHS Collaborative Research Group. Hypertension. (1992) 19:508–19. 10.1161/01.hyp.19.6.508 1592445

[20] Wieling W Krediet CT van Dijk N Linzer M Tschakovsky ME. Initial orthostatic hypotension: review of a forgotten condition. Clin Sci (Lond). (2007) 112:157–65. 10.1042/CS20060091 17199559

[21] Podoleanu C Maggi R Oddone D Solano A Donateo P Croci F. The hemodynamic pattern of the syndrome of delayed orthostatic hypotension. Interv Card Electrophysiol. (2009) 26:143–9. 10.1007/s10840-009-9429-0 19669396

[22] Berntson GG Thomas Bigger J Eckberg DL Grossman P Kaufmann PG Malik M. Heart rate variability: origins, methods, and interpretive caveats. Psychophysiology. (1997) 34:623–48. 10.1111/j.1469-8986.1997.tb02140.x 9401419

[23] Nguyen L Su S Nguyen HT. Effects of hyperglycemia on variability of RR, QT and corrected QT intervals in type 1 diabetic patients. Conf Proc IEEE Eng Med Biol Soc. (2013) 2013:1819–22. 10.1109/EMBC.2013.6609876 24110063

[24] Billman GE. Heart rate variability–a historical perspective. Front Physiol. (2011) 2:86. 10.3389/fphys.2011.00086 22144961 PMC3225923

[25] Shirai K Saiki A Nagayama D Tatsuno I Shimizu K Takahashi M. The role of monitoring arterial stiffness with cardio-ankle vascular index in the control of lifestyle-related diseases. Pulse (Basel). (2015) 3:118–33. 10.1159/000431235 26587461 PMC4646158

[26] Yao ST Hobbs JT Irvine WT. Ankle pressure measurement in arterial disease of the lower extremities. Br J Surg. (1969) 56:676–9. 10.1002/bjs.1800560910 5686989

[27] Xiao H Tan I Butlin M Li D Avolio AP. Arterial viscoelasticity: role in the dependency of pulse wave velocity on heart rate in conduit arteries. Am J Physiol Heart Circ Physiol. (2017) 312:1185–94. 10.1152/ajpheart.00849.2016 28364019

[28] Clegg A Young J Iliffe S Rikkert MO Rockwood K. Frailty in elderly people. Lancet. (2013) 381:752–62. 10.1016/S0140-6736(12)62167-9 23395245 PMC4098658

[29] Vijayan J Sharma VK. Neurogenic orthostatic hypotension–management update and role of droxidopa. Ther Clin Risk Manag. (2015) 11:915–23. 10.2147/TCRM.S68439 26089676 PMC4467737

[30] Pagani M Lombardi F Guzzetti S Rimoldi O Furlan R Pizzinelli P. Power spectral analysis of heart rate and arterial pressure variabilities as a marker of sympatho-vagal interaction in man and conscious dog. Circ Res. (1986) 59:178–93. 10.1161/01.res.59.2.178 2874900

[31] Akselrod S Gordon D Ubel FA Shannon DC Berger AC Cohen RJ. Power spectrum analysis of heart rate fluctuation: a quantitative. Science. (1981) 213:220–2. 10.1126/science.6166045 6166045

[32] Perini R Veicsteinas A. Heart rate variability and autonomic activity at rest and during exercise in various physiological conditions. Eur J Appl Physiol. (2003) 90:317–25. 10.1007/s00421-003-0953-9 13680241

[33] Pumprla J Howorka K Groves D Groves D Chester M Nolan J Functional assessment of heart rate variability: physiological basis and practical applications. Int J Cardiol. (2002) 84:1–14. 10.1016/s0167-5273(02)00057-8 12104056

[34] Shaffer F McCraty R Zerr CL A healthy heart is not a metronome: an integrative review of the heart's anatomy and heart rate variability. Front Psychol. (2014) 5:1040 10.3389/fpsyg.2014.01040 25324790 PMC4179748

[35] Shaffer F Ginsberg JP An overview of heart rate variability metrics and norms. Front Public Health. (2017) 5:258 10.3389/fpubh.2017.00258 29034226 PMC5624990

[36] Goldstein DS Bentho O Park MY Sharabi Y. Low-frequency power of heart rate variability is not a measure of cardiac sympathetic tone but may be a measure of modulation of cardiac autonomic outflows by baroreflexes. Exp Physiol. (2011) 96:1255–61. 10.1113/expphysiol.2010.056259 21890520 PMC3224799

[37] Houle MS Billman GE. Low-frequency component of the heart rate variability spectrum: a poor marker of sympathetic activity. Am J Physiol Heart Circ Physiol. (1999) 267:H215–23. 10.1152/ajpheart.1999.276.1.H215 9887035

[38] Hopf HB Skyschally A Heusch G Peters J. Low-frequency spectral power of heart rate variability is not a specific marker of cardiac sympathetic modulation. Anesthesiology. (1995) 82:609–19. 10.1097/00000542-199503000-00002 7879929

