# Supplementary material for: Study on the Relationship Between Orthostatic Hypotension and Heart Rate Variability, Pulse Wave Velocity Index, and Frailty Index in the Elderly: A Retrospective Observational Study
Source: Front Cardiovasc Med. 2020 Nov 27;7:603957. doi: 10.3389/fcvm.2020.603957 (PMC7728663; doi:10.3389/fcvm.2020.603957)
Supplement: Supplementary file 1 [file Data_Sheet_1.DOCX]

**Supplementary Table 1:** GFI Scale

| GFI Items | 1 | 0 | Score0/1 |
| --- | --- | --- | --- |
| Physical field  1.Can't shopping alone | Yes□ | No□ |  |
| 2.Can't walk outdoors alone | Yes□ | No□ |  |
| 3.Can't put/take off clothes alone | Yes□ | No□ |  |
| 4.Can't go to the bathroom alone | Yes□ | No□ |  |
| 5.Always tired | Yes□ | No□ |  |
| 6.Visual impairment affects life | Yes□ | No□ |  |
| 7.Hearing loss affects life | Yes□ | No□ |  |
| 8.Weight loss | Yes□ | No□ |  |
| 9.Four or more prescription drugs required | Yes□ | No□ |  |
| Cognitive field  10.Marked decline in memory affects life | Yes□ | No□ |  |
| Social field  11.Social networking barriers | Yes□ | No□ |  |
| 12.Not care about others | Yes□ | No□ |  |
| 13.Not helping others  Psychological field | Yes□ | No□ |  |
| 14.Can't stay calm and relax  15.Often nervous or depressed | Yes□  Yes□ | No□  No□  Total points |  |

**Supplementary Table 2:** Evaluation of FI Projects

|  | 1 | 0.5 | 0 | |
| --- | --- | --- | --- | --- |
| Self-care capacity（6 Items）  1.Cooking | No□ |  | Completed independently□ | |
| 2.Shopping | No□ |  | Completed independently□ | |
| 3.Do the housework | No□ |  | Completed independently□ | |
| 4.Bathing | No□ |  | Completed independently□ | |
| 5.Urine control | No□ |  | Yes□ | |
| 6.Outdoor walk | Completed independently□ |  | No□ | |
| Cognitive ability（1Item）   1. MMSE Scale Rating Illness（16Items） | <10□ | 10-25□ | >25□ | |
| 8.Allergies other than food | Yes□ |  | No□ | |
| 9.Asthma | Yes□ |  | No□ | |
| 10.Degenerative joints  11.Rheumatism  12.Hypertension  13.Diabetes  14.Migraines  15.Chronic bronchitis  16.Sinusitis  17.Epilepsy  18.Heart disease  19.Tumors  20.Gastrointestinal diseases  21.Cerebrovascular diseases  22.Alzheimer's disease or other dementia  23.Cataracts | Yes□  Yes□  Yes□  Yes□  Yes□  Yes□  Yes□  Yes□  Yes□  Yes□  Yes□  Yes□  Yes□  Yes□ |  | No□  No□  No□  No□  No□  No□  No□  No□  No□  No□  No□  No□  No□  No□ | |
| Health evaluation（4 Items）  24.Hearing impairment affects life  25.Visual impairment affects life  26.Serious illness in the past year(seriously ill or critically ill)  27.Four or more prescription drugs required | Yes□  Yes□  Yes□  Yes□ |  | No□  No□  No□  No□ | |
| Mental health（2 Items）   1. Often nervous or depressed   29.Not interested in things  Other（1 Item）   1. Unconscious weight loss 5 kg(1 year)   Total Score/FI | Yes□  Yes□  Yes□ |  | | No□  No□  No□ |
